# Clinical Perspectives on Surgical Reconstruction of Eccentric Tumors at the Distal Femur with Unicondylar Resection

**DOI:** 10.1111/os.14119

**Published:** 2024-06-24

**Authors:** Xin Hu, Chende Wang, Yi Zeng, Xiao Yang, Li Min

**Affiliations:** ^1^ Department of Orthopedic Surgery and Orthopedic Research Institute, West China Hospital Sichuan University Chengdu China; ^2^ Model Worker and Craftsman Talent Innovation Workshop of Sichuan Province, West China Hospital Sichuan University Chengdu China; ^3^ National Engineering Research Center for Biomaterials Sichuan University Chengdu China; ^4^ Provincial Engineering Research Center for Biomaterials Genome of Sichuan Sichuan University Chengdu China

**Keywords:** Allograft transplantation, Osteoarthritis, Prosthesis, Unicompartmental knee arthroplasty, Unicondylar resection

## Abstract

The distal femur is one of the most common sites for primary bone tumors. As the tumor progresses and bone destruction worsens, it can severely affect knee function and even pose a threat to life. In cases where only one condyle is affected and requires resection, preserving the healthy contralateral condyle can substantially enhance the biomechanics of the knee. Furthermore, preserving bone stock may enable future salvage procedures in the event of initial surgery failure, be it from fractures or osteoarthritis. Distal femoral unicondyle resection can offer better functional outcomes in select cases. However, it is essential to prioritize oncological safety with adequate margins over short‐term knee function. Currently, the primary methods for reconstruction after the excision of a unicondylar tumor include allograft transplantation (bi‐ or uni‐condylar) and prosthetic or allograft‐prosthesis composite replacement (APC). However, there is currently some controversy regarding the optimal surgical reconstruction method, and a consensus within the academic community has yet to be reached. Moreover, due to the rarity of bone tumors, extensive clinical data from a single center is limited. Current studies are mainly retrospective and single‐center, lacking sufficient cases and follow‐up duration. This article reviews surgical reconstruction after solitary condylar excision in distal femoral tumors. It summarizes, compares, and analyzes mainstream reconstruction methods, exploring their technical details and clinical outcomes to highlight their potential in bone oncology.

## Graphical abstract

The distal femur hosts common primary bone tumors, impacting knee function and life. Preserving the healthy contralateral condyle and ligaments enhances knee biomechanics. Reconstruction methods post‐unicondylar excision vary, including allograft transplantation and prosthetic replacement. This review compares these methods, aiming to guide optimal surgical reconstruction for distal femoral tumors.

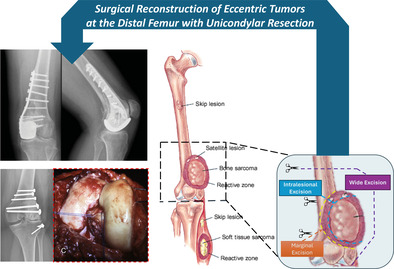

## Introduction

Malignant or benign bone tumors often emerge in the proximity of joints, notably prevalent in the distal femur.^1^ For instance, the predominant primary malignant bone tumor, osteosarcoma, manifests around 85% of cases in the distal femur, proximal tibia, and proximal humerus.^2^ A common benign yet invasive bone tumor, giant cell tumor of bone (GCTB), is observed in this region in approximately 33.8% of cases.^3^ With advancements in modern chemotherapy, radiotherapy, imaging technology, and surgical techniques, limb salvage surgery employing en bloc resection with clear margins has emerged as the predominant approach for treating bone tumors around the knee joint.^4^ The surgical management of benign tumors typically involves curettage, with or without adjuvant therapies, proving effective in most cases. However, the treatment approach shifts for aggressive stage III benign lesions, such as giant cell tumors, or malignant bone tumors, requiring en bloc resections to achieve local control. Addressing segmental osteoarticular bone defects following en bloc resection poses a notable surgical challenge due to the imperative to restore stability, motion, and function in the weight‐bearing joint. While megaprosthetic implant replacement stands out as the most common reconstruction method, it comes with a heightened complication rate compared to total knee arthroplasty (TKA), encompassing concerns like septic loosening and aseptic loosening.^5^, ^6^

A distinctive circumstance arises when tumors are confined to a single condyle with well‐defined margins. Opting for a unicondyle resection ensures broad, adequate margins with minimal bone sacrifice. This diversified range of surgical choices enhances flexibility in selecting appropriate reconstruction methods. The preservation of the unaffected contralateral condyle, along with crucial ligamentous structures instrumental in maintaining knee joint stability, markedly enhances knee biomechanics. Furthermore, judicious conservation of bone stock not only offers prospects for subsequent salvage interventions, including megaprosthesis, conventional TKA, unicompartmental knee prosthesis, or emerging osteoarticular allograft transplantation (UOA) modalities, but also proves indispensable in mitigating potential complications arising from the initial surgical endeavor, whether attributable to fracture or symptomatic knee osteoarthritis. This holds heightened significance, particularly among youthful, active patients with elevated aspirations for both ambulation and nuanced functional restoration. Due to the aforementioned considerations, unicondyle resection and reconstruction are theoretically posited as the optimal approach for addressing both benign and malignant tumors confined to a single condyle of the knee joint.

However, the array of surgical reconstructions following unicondyle resection is notably diverse, yielding varied treatment outcomes. Principal modes of reconstruction encompass allograft transplantation (bi‐ or uni‐condylar) and prosthetic or composite (allograft plus prosthesis) replacement.^7^, ^8^, ^9^, ^10^, ^11^ Therefore, ongoing debate persists regarding the optimal surgical reconstruction method, lacking a definitive consensus within the academic sphere.^7^ Furthermore, the rarity of distal femoral condylar bone tumor cases has hindered the collection of comprehensive clinical data from a single tumor center. Existing studies primarily consist of single‐center retrospective analyses, lacking sufficient case numbers and follow‐up duration. Thus, discrepancies in patient inclusion/exclusion criteria, follow‐up durations, reconstruction methodologies, and outcome assessment criteria contribute to the challenge of formulating a unified understanding of the most effective reconstruction strategy following unicondyle resection for distal femoral tumors. Hence, the objective of this manuscript is to meticulously review and analyze extant literature on surgical reconstructions subsequent to unicondyle resection for distal femoral tumors. It aspires to succinctly outline, compare, and analyze the technical nuances of prevalent reconstruction methods, probe into the clinical prognosis associated with diverse reconstruction approaches, and thereby underscore their potential contributions to the evolving landscape of bone oncology.

## Literature Search Methodology

### Inclusion and Exclusion Criteria

The inclusion criteria were defined as follows: (i) study type—original articles, clinical articles, review articles, guidelines, editorials, commentaries, surgical techniques, and case reports; (ii) study subjects—various surgical reconstruction methods and related clinical follow‐up studies after distal femoral condyle resection; and (iii) observation indexes—functional outcomes post‐surgical reconstruction, pain control, oncology outcomes, and imaging outcomes. The exclusion criteria were delineated as follows: (i) repeated publication of identical population data; (ii) articles lacking clearly described original data; (iii) research on reconstruction following total distal femoral condyle resection; (iv) research on reconstruction after proximal tibial tumor resection; and (v) research on unicompartmental knee arthroplasty for osteoarthritis.

### Study Retrieval and Keywords

**FIGURE 1:**
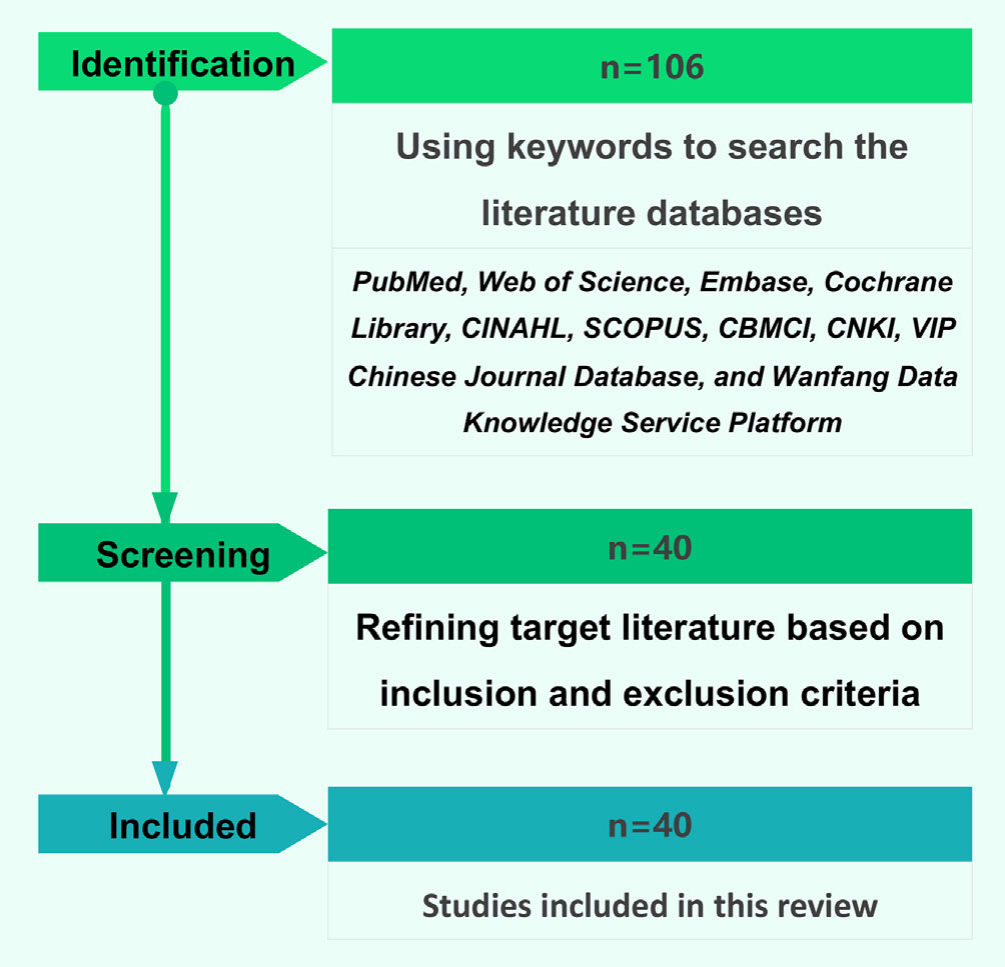
Flowchart of literature selection.

A total of 10 databases, including PubMed, Web of Science, Embase, Cochrane Library, CINAHL, SCOPUS, CBMCI, CNKI (China National Knowledge Infrastructure), VIP Chinese Journal Database, and Wanfang Data Knowledge Service Platform, were utilized for study retrieval from database establishment to February 2024. The following keywords and their synonyms were employed for the search: distal femur, tumor, surgery, and unicondylar. The retrieval and screening process are illustrated in Figure 1.

## Surgical Margins and Recurrence Risk in Unicondyle Tumor Excision

**FIGURE 2:**
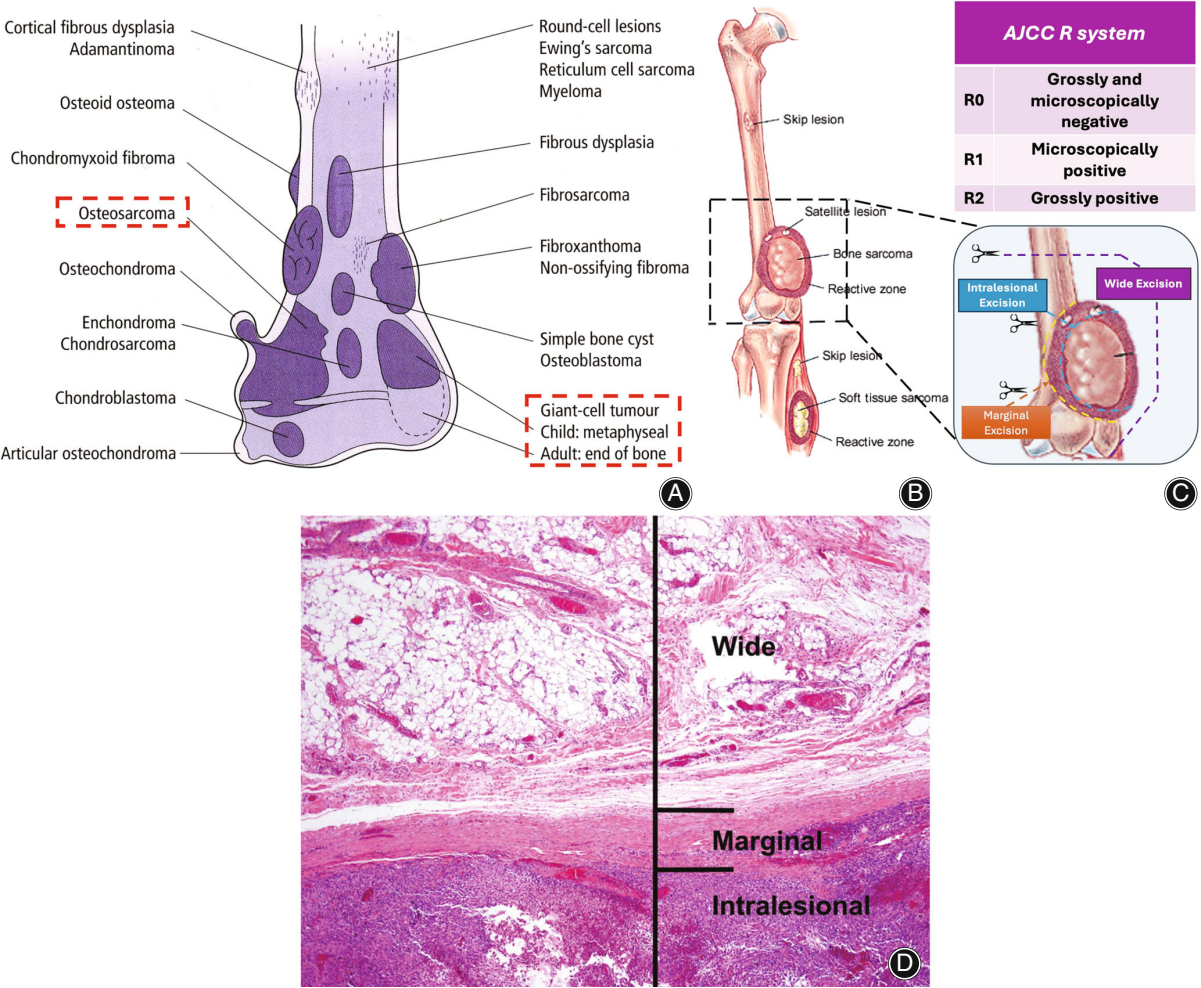
Illustrates the eccentrically growing tumors at the distal end of the femur, focusing on single‐compartment resection and the determination of surgical margins. (A) Depicts the common anatomical location of bone tumors, with many tumors exhibiting eccentric growth. Reprinted with permission from Plant and Canno*n* ^43^ (B) Provides a schematic representation of the MSTS surgical margin assessment system for distal femoral bone tumors, showcasing the resection ranges for wide excision, marginal excision, and intralesional excision. It also describes the biologic growth pattern of osteosarcomas, emphasizing the radial growth locally, the formation of a pseudocapsular layer known as the “reactive zone,” and the presence of microscopic extensions called “satellites.” Skip metastases within the same bone (intraosseous) or across adjacent joints (transarticular) are explained, along with distant metastases to the lungs and other bones. Reprinted with permission from Bickels *et al*.^44^ (C) Outlines the scope of RO/1/2 resection in the AJCC R system. (D) Displays a photomicrograph of the surgical resection margin of a sarcoma, indicating MSTS margin classifications (H&E, 40×). Reprinted with permission from Cates *et al*.^45^

Tumor recurrence post‐excision is primarily influenced by the completeness of surgical excision, particularly the adequacy of surgical margins, alongside other factors such as tumor type, staging, postoperative treatment, and patient immune status (Figure 2A). Currently, the most commonly utilized surgical margin systems include the Musculoskeletal Tumor Society's (MSTS) surgical margin assessment system (Figure 2B),^12^ and the Union for International Cancer Control's (UICC) R0/R1/R2 resection standards (Figure 2C).^13^, ^14^ In studies focusing on distal femoral unicondyle tumor excision, there is concern regarding an increased risk of local recurrence associated with more constrained resections. However, all studies consistently reported recurrence rates ranging from 4% to 6%, irrespective of the resection method employed, thereby failing to substantiate this hypothesis.^7^, ^15^, ^16^, ^17^, ^18^ The assertion that there is an increased risk of recurrence following unicondyle resection lacks support in the literature. For instance, there is no significant difference in the postoperative recurrence risk between autologous bone reconstruction following bicondylar resection and autologous bone reconstruction after unicondyle resection. Local recurrence rates for bicondylar osteoarticular allograft reconstruction varied from 0%^15^ to 8%.^19^ In the case of unicondylar osteoarticular allograft reconstruction, rates ranged from 0%^20^ to 5%.^17^ However, it is essential to avoid prioritizing short‐term knee function over oncological safety with adequate margins in unicondyle tumor excision and reconstruction. This approach emphasizes accurate tumor excision with appropriate margins. The literature suggests that for low‐grade bone sarcomas like chondrosarcoma, a 10‐mm tumor‐free bone resection margin is recommended. For high‐grade sarcomas such as osteosarcoma and Ewing sarcoma without effective preoperative treatment, a 30‐mm margin is recommended; with effective preoperative treatment, a 20‐mm margin suffices.

**FIGURE 3:**
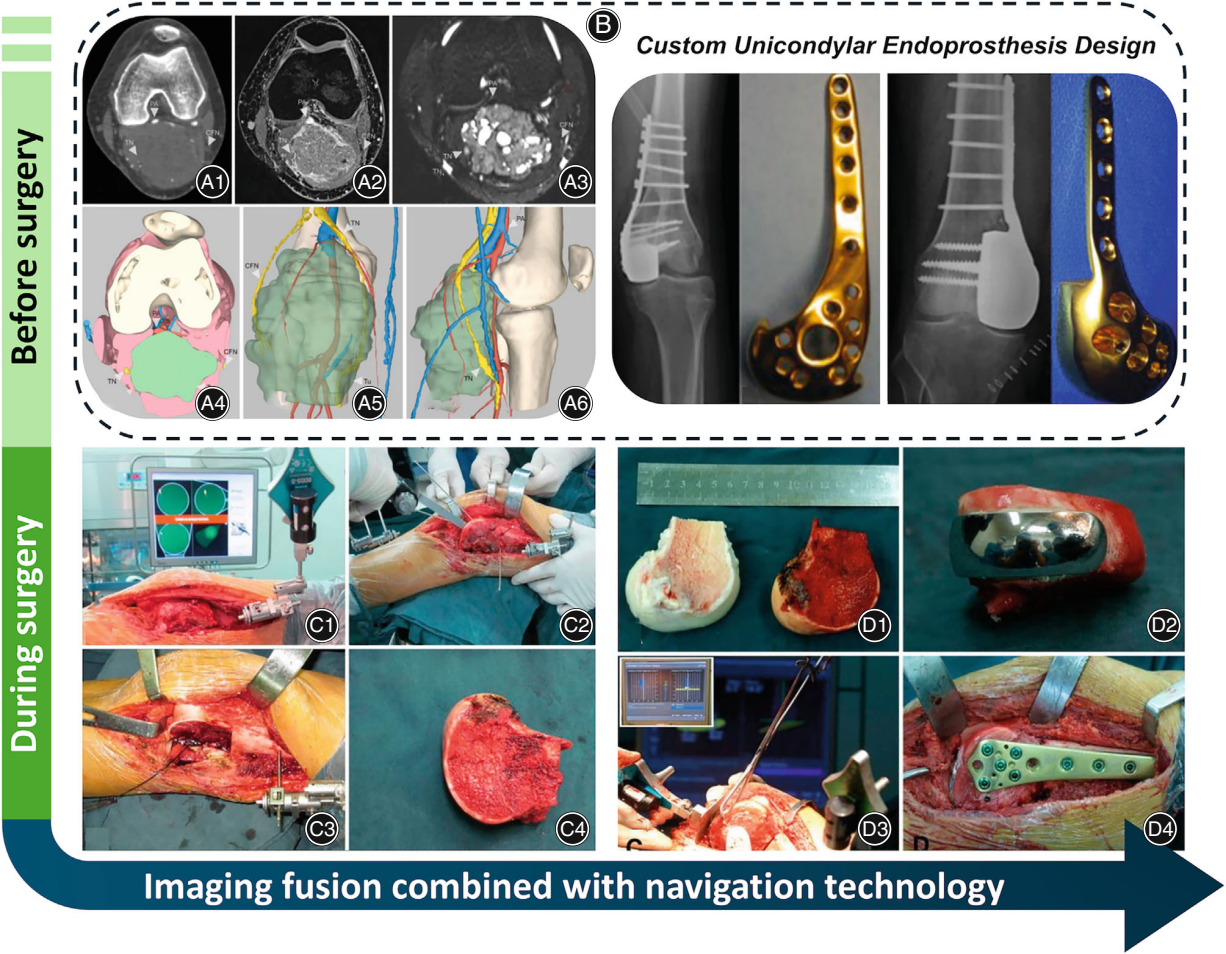
Precision in surgical margin determination achieved through multimodal imaging fusion and intraoperative navigation. (A) Illustrates personalized surgical planning for soft tissue sarcoma in the popliteal fossa using an innovative 3D imaging technique. Classic images and three‐dimensional reconstructions for a popliteal fossa synovial sarcoma patient include CT angiography (A1), T1‐weighted MRI (A2), MR hydrography (A3), and 3D reconstruction images (A4–A6). Reprinted with permission from Fang *et al*.^22^ (B) Custom unicondylar endoprosthesis design utilizing 3D modeling software. Reprinted with permission from Ippolito *et al*.^10^ (C) These images illustrate tumor resection with computer‐assisted navigation. (C1) A femoral tracker was applied for calibration, aligning operative anatomy precisely with the virtual image. (C2) Navigated tools marked the intended bone resection plane with a K‐wire. (C3) An oscillating saw removed the lateral femoral condyle. (C4) En bloc tumor resection ensured a wide margin. (D) Depicting the unicondylar osteoallograft prosthesis reconstruction using computer‐assisted navigation. (D1) The osteoarticular allograft was tailored to fit the bone defect. (D2) The cemented femoral component was affixed, forming the unicondylar osteoallograft prosthesis composite. (D3) Navigation enabled precise implantation, ensuring congruency of the articular surface. The insert (upper left) shows mechanical alignment verification. (D4) The composite was secured to the host bone with a locking plate. Reprinted with permission from Fan *et al*.^9^

Additionally, based on research on unicondyle tumor excision, to effectively reduce the risk of postoperative recurrence, the following strategies could be considered: accurate surgical margin determination through multimodal imaging fusion and intraoperative navigation. Three‐dimensional multi‐modality images (3DMMI) integrate diverse imaging data to present comprehensive 3D images on computers.^21^ Leveraging 3DMMI technology allows surgeons to accurately assess tumor boundaries preoperatively, facilitating precise bone resection planning with adequate surgical margins. Fang *et al*.^22^ investigated the efficacy of this technique for preoperative planning of soft tissue sarcomas (STS) in the popliteal fossa. In their prospective pilot study of 27 patients, 3DMMI showed a trend toward lower inadvertent positive margin rates (Figure 3A), alongside significant reductions in hospital stays, operative time, and blood loss compared to traditional methods. Additionally, in combination with 3DMMI technology, medical image analysis software can generate customized unicondylar endoprosthesis designs for reconstruction after distal femoral unicondyle tumor excision, ensuring precise matching with the tibial joint surface (Figure 3B). Furthermore, Fan *et al*.'s study^9^ employed a computer‐assisted navigation system (Stryker Pacific Ltd., Hong Kong, China) for precise unicondyle tumor resection and reconstruction, ensuring surgical precision. Surgeons using navigation systems can better replicate their surgical plans, enhancing the accuracy of bone tumor surgery (Figure 3C,D).^23^

A favorable response to chemotherapy is a key factor guiding patients toward choosing unicondyle tumor excision and reconstruction. While an insufficient surgical margin can lead to recurrence, effective chemotherapy can mitigate the recurrence rate to a more favorable level, even in cases with inadequate margins. Gherlinzoni *et al*.^24^ investigated the impact of chemotherapy‐induced necrosis and surgical margins on local recurrence in osteosarcoma cases. The study, encompassing 355 cases of high‐grade osteosarcoma with 237 patients undergoing limb‐sparing treatment, identified the most crucial factors influencing recurrence as post‐chemotherapy tissue necrosis, followed by surgical margins. Importantly, both factors were independent prognostic indicators for recurrence. In instances where chemotherapy response was favorable and surgical margins were extensive, the recurrence rate was less than 1%.

## Allograft Transplantation

Following distal femoral tumor resection, the most common reconstruction method involves megaendoprosthetic replacement, which requires resection of the unaffected condyle with contralateral joint surface. This procedure typically leads to substantial bone loss and carries a high risk of complications, such as prosthetic loosening (6% to 84%), periprosthetic infection (7% to 41%), and periprosthetic fractures (5% to 15%).^25^, ^26^ Allograft transplantation is another primary reconstruction method documented in the literature. Initially introduced as bicondylar osteoarticular allograft (BOA) transplantation, it is known for its demanding nature and biologically oriented knee reconstruction approach. However, it is associated with significant bone loss and various complications, including allograft infection (10% to 23%), allograft fracture (17% to 31%), nonunion (17% to 20%), and cartilage degeneration (25% to 31%).^15^, ^27^, ^28^

Furthermore, autologous tumor‐bearing bone inactivation replantation refers to the process of physically or chemically deactivating residual autologous bone following tumor resection (utilizing methods such as pasteurization, external radiation, cryotherapy with liquid nitrogen, or immersion in anhydrous ethanol).^29^, ^30^, ^31^, ^32^ This approach offers advantages in terms of immunological compatibility and anatomical conformity with host bone, presenting a lower incidence of long‐term complications compared to megaendoprosthetic reconstruction as a biological reconstruction strategy.^25^, ^26^ However, autologous tumor bone inactivation replantation encounters a significant paradox: complete tumor eradication often results in substantial loss of the mechanical integrity of autologous bone, thereby escalating the risk of postoperative fractures and impaired bone healing, while incomplete inactivation can lead to postoperative tumor recurrence. Thus, the technique's application is debated, and there is currently no literature on inactivation replantation following distal femoral single‐condyle tumor resection.

**FIGURE 4:**
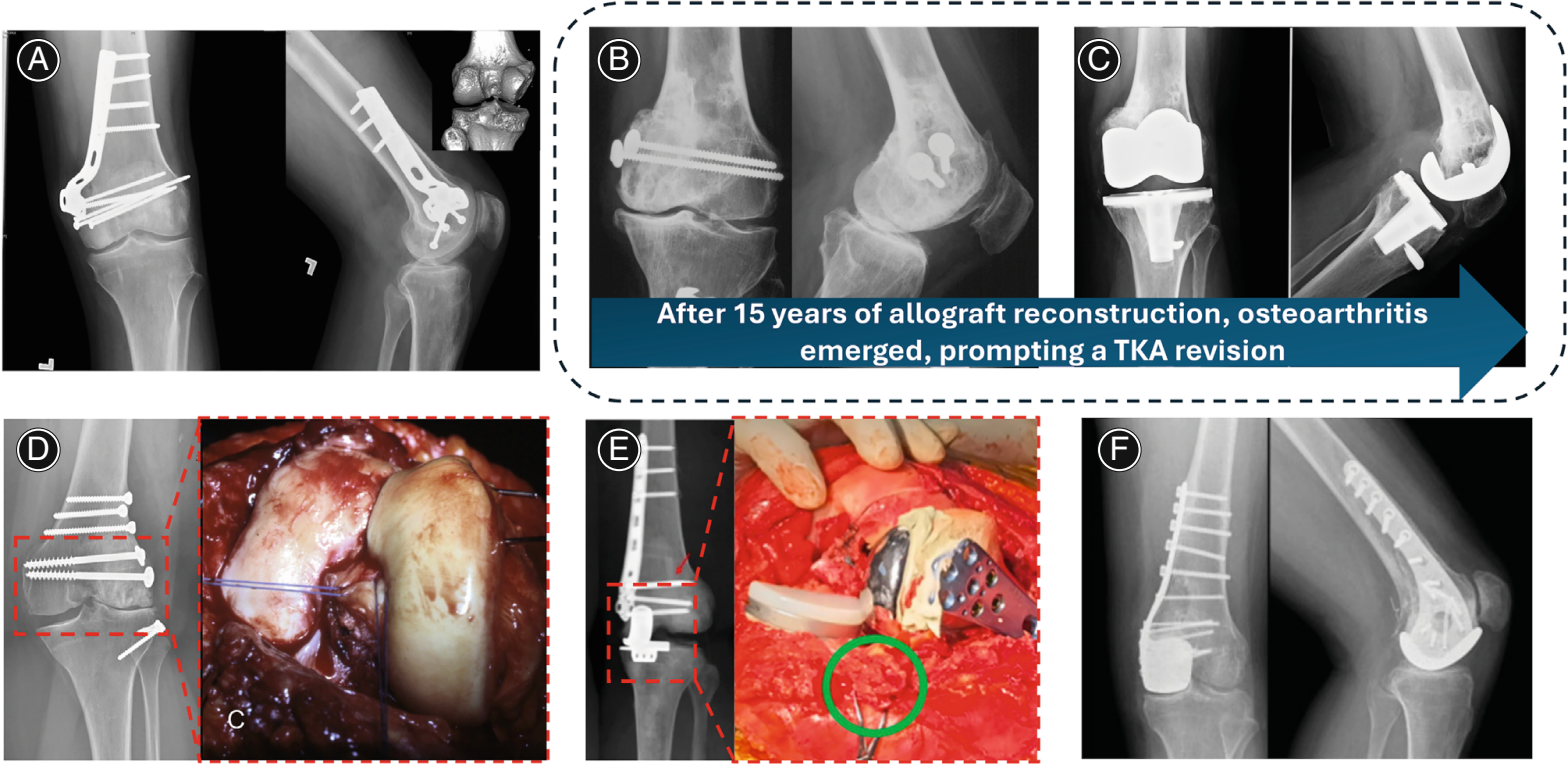
Surgical reconstruction following resection of distal femoral unicondylar tumors. (A) Implementation of large osteochondral allografts for the reconstruction of traumatic uncontained distal femoral defects. A CT three‐dimensional reconstruction reveals a substantial osteochondral defect in the patient's medial femoral condyle. Anteroposterior and lateral radiographs taken at 2 years post‐op demonstrate the well‐positioned allograft with bridging callus across the fracture site and no degenerative changes. Reprinted with permission from Fagan *et al*.^9^ (B) Severe osteoarthritis manifested in a medial femoral condyle allograft (a) 15 years post‐initial allograft reconstruction. Due to adequate bone stock preservation, the patient's knee underwent reconstruction with a posterior‐stabilized prosthesis. (C) X‐rays, taken 7 years later, illustrate the well‐positioned implant and indicate a favorable clinical prognosis. (D) Giant cell tumor of the lateral femoral condyle in a 21‐year‐old female. The condyle was resected, and intraoperative images depict joint line reconstruction with ligament reattachment. The allograft shows robust integration 23 years postoperatively. Reprinted with permission from Bianchi *et al*.^18^ (E) A 39‐year‐old female with a Giant Cell Tumor and pathological fracture in the left distal femur underwent unicondylar osteoallograft prosthesis composite reconstruction. The autologous meniscus was preserved (green circle), and UKA reconstruction was performed on the tibial side. Reprinted with permission from Liu *et al*.^8^ (F) A 17‐year‐old girl with osteosarcoma underwent complex medial femoral condyle resection and osteoarticular allograft reconstruction. Fourteen months later, an allograft fracture required a successful revision with custom unicondylar hemiarthroplasty, resulting in excellent clinical function. Reprinted with permission from Ippolito *et al*.^10^

Preserving the unaffected condyle offers potential benefits in knee biomechanics enhancement. Therefore, UOA transplantation has emerged as an alternative for distal femur or proximal tibia reconstruction after tumor resections or substantial post‐traumatic bone defects around the knee (Figure 4A). As early as 1985, Campanacci *et al*.^33^ introduced the concept of unicondyle resections with reconstruction using a patellar autograft, and similarly, Farooque and Sharma^34^ reported favorable outcomes employing the same technique. In recent years, deep‐frozen allografts have supplanted autografts, mitigating harvest‐related morbidity (Figure 4B). However, the unique preservation method of frozen unicondylar osteoarticular allografts has been associated with the degeneration of chondrocytes, potentially influenced by factors like processing techniques involving cytoprotective agents and the rate of slow freezing. This presents a significant limitation to the clinical application of UOA. Moreover, challenges arise from the difficulty in precisely matching the size of the unicondylar osteoarticular allograft, leading to malalignment and accelerating the degenerative process. The stability of ligamentous repair is another factor contributing to potential cartilage degeneration. These considerations underscore the critical hurdles that impede the widespread use of UOA in clinical practice (Figure 4C,D). For example, Bianchi *et al*.^20^ reported on the outcomes of 10 unicondylar osteoarticular allografts. Among these, two demonstrated excellent function, five had good function, and three exhibited fair function, with a minimum follow‐up of 4 years (average, 11 years). However, radiographically, five patients showed mild degenerative changes, while five others had severe degenerative changes (Figure 4E). In a study with an average clinical follow‐up of 11 years involving 40 unicondylar osteoarticular allograft reconstructions, Muscolo *et al*.^19^ reported that the mean radiographic score for the 33 surviving allografts was 89%, with an average functional score of 27 points. However, 39% (13) of the remaining allografts exhibited articular deterioration, including 18% (six) with joint narrowing of 2 mm, 9% (three) with joint narrowing of 4 mm, and 12% (four) showing subchondral bone collapse. In summary, osteoarticular allograft reconstructions are associated with elevated rates of mechanical complications. Despite limited comparative studies with alternative techniques, we believe that the perceived risk of mechanical failure does not justify the routine use of osteoarticular allografts for reconstructing large joints following tumor resection.

## Unicondylar Osteoallograft Prosthesis Composite Reconstruction

The absence of viable chondrocytes in frozen osteoarticular allografts leads to progressive degeneration of the articular surface in both bicondylar and unicondylar osteoarticular allograft reconstructions. Additionally, challenges in achieving optimal matching between allograft and host bone contribute to the reported rates of moderate and severe joint degeneration ranging from 31% to 72%. Although unicondylar osteoarticular (UOA) reconstruction effectively preserves the native anatomy, it often leads to suboptimal knee function and dissatisfaction. Therefore, some scholars have explored new surgical reconstruction approaches by combining UOA surgery with unicompartmental knee arthroplasty (UKA). This hybrid approach aims to achieve the benefits of biologic reconstruction with UOA and protect joint surfaces and subchondral bone with UKA, thereby reducing the risk of long‐term joint degeneration postoperatively.

UKA is an effective treatment for medial compartment osteoarthritis, providing superior knee function, recovery, and fewer complications than TKA. In Liu *et al*.'s study,^8^ combining UOA reconstruction with UKA for treating distal femur giant cell tumors (GCTs) was evaluated in 73 patients from 2000 to 2015. UKA patients showed improved knee function with higher Knee society scores (KSS) and lower western ontario and McMaster universities arthritis (WOMAC) scores compared to alternative treatments. This combination is suggested to enhance knee function, slow osteoarthritis progression, and is favorable for GCT patients seeking joint preservation and improved function. In a similar retrospective study by Fan *et al*.,^9^ 12 patients underwent unicondylar osteoallograft prosthesis composite reconstruction following tumor resection. The results showed a promising survival rate with minimal complications, as 10 out of 12 reconstructions remained intact at the last follow‐up. Functional and radiographic assessments indicated few significant complications and short‐term preservation of tibial cartilage. However, significant differences between studies emerged in the intraoperative intricacies of UKA reconstruction. Liu *et al*.^8^ incorporated an extramedullary tibial resection guide and proximal tibial resection of the allograft. The tibial components were secured with polymethyl methacrylate (PMMA) bone cement, and a mobile polyethylene bearing was inserted. In contrast, Fan *et al*.'s^9^ simplified approach omitted the handling of tibial components, avoiding the replacement of the opposing tibial plateau with a metallic prosthesis. This streamlined technique prevented potential tumor cell contamination, retained cartilage, and reduced operation time, resulting in satisfactory long‐term functional outcomes. Noteworthy is the extended operation time and heightened intraoperative bleeding in unicondylar osteoallograft prosthesis composite reconstruction compared to conventional unicondylar osteoarticular allograft reconstruction. Nevertheless, the increased operation time remained relatively short, notably shorter than that of regular UKA. The larger surgical field facilitated enhanced exposure, mitigating the complexities associated with traditional UKA.

## Custom Unicondylar Hemiarthroplasty

Following distal femoral unicondyle tumor resection, the inherent threats of complications tied to allogeneic bone transplantation, whether executed through simple osteoarticular allograft transplantation or unicondylar osteoallograft prosthesis composite reconstruction, encompass challenges like limited graft availability, disease transmission, infection, fractures, and nonunion.^9^, ^18^, ^35^, ^36^, ^37^ Achieving precise matching between the graft and host bone, particularly when stringent knee joint surface requirements are involved, heightens the difficulty of selecting and shaping allografts for an aesthetically matched appearance. To mitigate these risks, prosthetic implants offer advantages such as a less intricate technique, shelf availability, and early weight‐bearing capabilities, positioning them as the gold standard for such resections.^38^ Nevertheless, a drawback of megaendoprosthetic joint arthroplasty is the sacrifice of the corresponding reciprocal side of the joint. Recent studies underscore notable failure rates in megaendoprosthetic reconstruction, attributed to both mechanical issues like loosening, implant breakage, and periprosthetic fractures, and non‐mechanical problems, predominantly infections.^39^

Scholars have recently advocated for the use of customized unicondyle endoprostheses in post‐resection reconstruction. This approach modifies implant design to avoid complications associated with allogeneic bone transplantation. The tailored prosthesis precisely matches the host bone, reducing the risk of joint degeneration due to mismatch. Importantly, custom unicondyle prostheses preserve the healthy side of the femoral condyle, potentially maintaining higher functionality and stability by retaining native anatomy. In addition to functional benefits, customized unicondyle hemiarthroplasty shows promise for preserving more bone stock in case revision surgery is needed in the future. While cases of custom unicondylar hemiarthroplasty after traumatic bone loss have been reported, there is a lack of comprehensive literature on its application in tumor patients, although initial outcomes are promising.^40^, ^41^

Ippolito *et al*.^10^ introduced the use of customized unicondyle prostheses for reconstruction following distal femural tumor resection. Prostheses were utilized as a secondary procedure in two cases due to initial reconstruction failure and as the primary reconstruction method in one case. The mean MSTS scores at a mean follow‐up of 45 months were 26.7. All patients successfully regained full weight‐bearing capacity, resumed activities of daily living, and achieved a functional range of motion. To ensure stability of the knee joint, medial collateral ligament (MCL) reconstruction was conducted using a semitendinosus autograft. Soft tissue coverage was achieved by releasing the distal end of the sartorius and suturing it onto the vastus medialis. For reconstruction of the lateral femoral condyle, soft tissue coverage was provided with a gastrocnemius flap (Figure 4F). Similarly, in the study by Pala *et al*.,^42^ a retrospective analysis compared the short‐term efficacy of 3D‐printed custom distal femoral unicondyle prostheses and tumor knee arthroplasty in 26 patients with eccentric stage III giant cell tumors of the distal femur after tumor resection. The 3D‐printed group showed shorter surgical time, less blood loss, and significantly higher MSTS scores from 3 to 24 months postoperatively compared to the total knee arthroplasty group. The study suggests that 3D‐printed custom prostheses provide better short‐term functional results than total knee arthroplasty.

Surgical reconstruction post distal femoral unicondyle tumor resection can be achieved using osteoarticular allograft transplantation, combined allograft and prosthesis reconstruction, or custom prosthesis reconstruction. Allograft transplantation, though effective, has uncertain long‐term stability due to allograft‐related complications. Combined allograft and prosthesis reconstruction aims to balance functionality and stability but also faces unresolved allograft‐related challenges. Advances in additive manufacturing have spurred interest in 3D‐printed custom unicondyle prostheses for reconstruction. These prostheses provide anatomical conformity, minimizing joint degeneration risk, and their porous structure promotes osteointegration and long‐term stability. However, there are currently limited reports on such prostheses, and the long‐term functional prognosis of 3D printed customized unicondyle prostheses requires further clinical research with longer follow‐up periods and a larger number of cases to validate.

## Conclusion

Resection of the distal femoral unicondyle preserves the intact condyle and its supportive soft tissue structures, which are crucial for knee joint stability. This approach may be suitable for certain cases of primary bone tumors. However, ensuring oncological safety with adequate margins is paramount, prioritizing this over short‐term knee function outcomes when using this technique. Additionally, there are various reconstruction methods available following distal femoral unicondyle tumor resection. Among these, 3D‐printed customized prosthetic reconstruction is gaining traction due to its ability to precisely match bone defect morphology and promote bone integration with its porous surface structure. This trend is gradually replacing allogeneic bone transplantation, thus mitigating the complications associated with allografts.

## Author Contributions

Xin Hu, conceptualization, data curation, methodology, visualization, writing—original draft, writing—review and editing; Chende Wang, methodology, writing—review and editing; Yi Zeng, conceptualization, data curation, methodology, visualization, writing—original draft, writing—review and editing, supervision, and funding acquisition; Xiao Yang, conceptualization, Data curation, methodology, writing—review and editing, supervision; and Li Min, conceptualization, data curation, methodology, visualization, writing—original draft, writing—review and editing, supervision, funding acquisition.

## Conflict of Interest Statement

The authors declare that they have no conflicts of interest.

## Data Availability

The datasets used and/or analyzed during the current study are available from the corresponding author on reasonable request.
